# Responses of Autumn Phenology to Climate Change and the Correlations of Plant Hormone Regulation

**DOI:** 10.1038/s41598-020-65704-8

**Published:** 2020-06-03

**Authors:** Shixi Zhang, Junhu Dai, Quansheng Ge

**Affiliations:** Key Laboratory of Land Surface Pattern and Simulation, Institute of Geographic Sciences and Natural Resources Research, CAS, Beijing, 100101 China

**Keywords:** Climate sciences, Environmental sciences

## Abstract

Current understanding of autumn phenological responses to climate change in deciduous tree species remains limited, mainly due to the difficulties in defining autumn events and the lack of knowledge about its mechanism. Here we applied a method based on measuring chlorophyll A (Chla) content in leaf tissue during the entire autumn senescence processes to appropriately quantify autumn phenological processes. Beginning of leaf coloring could be defined as when about 50% of the Chl was lost. End of leaf coloring could be defined as when about 95% of the Chl was lost. Then the mechanism behind the timing of autumn senescence responses to climate change through hormone regulation was studied for the first time. Four dominate deciduous tree species with representative senescence type (*Salix babylonica*, *Ginkgo biloba*, *Acer mono*, *Cotinus coggygria*) were chosen as the subject of study. Variations in climate factors (temperature, day length, precipitation, humidity) were recorded and nine major endogenous hormones (IAA, IPA, ZR, DHZR, GA_3_, GA_4_, ABA, MeJA, BR) in leaf tissues were monitored during the entire autumn senescence processes. The experimental results verified temperature and day length are the major climate factors affecting autumn phenology. Low temperature and short day length could result in the decrease of ZR level and the increase of ABA level in leaf tissue, which directly trigger/promote senescence. Meanwhile, low temperature and short day length could cause the decrease of MeJA level and the increase of GA_3_ and GA_4_ level, which regulate the timing of autumn senescence indirectly through ZR, ABA, and IAA. Our study improves the understanding of autumn phenological response to climate change in deciduous trees.

## Introduction

Climate change has caused dramatic plant phenological shifts with substantial ecological and environmental consequences^1^. Changes in autumn phenology (i.e. timing of leaf coloration and leaf drop) alter the reproductive capacity of individuals and affect the net productivity of ecosystems^2^. Despite the importance, autumn phenology has not received sufficient attention comparing to spring phenology since it is more challenging to understand^3^.

To date, important progresses in understanding the effects of climate change on the timing of autumn leaf senescence have been made. Temperature and photoperiod appear to be the primary climate variables that regulate autumn phenology of deciduous trees^4^. For some species, leaf senescence can be driven more by temperature than photoperiod^5^. On average, long-term observational studies indicate leaf senescence is delayed by increasing temperature. Precipitation were also noted to have influence on autumn senescence for silver birch (*Betula pendula*), with a greater number of rainless days in September coinciding with an advance in the timing of senescence^6^. In contrast to spring phenology, the mechanism of the relationships between autumn phenology and climate factors is largely unelucidated due to the complexity of the driving factors of autumn phenology and the protracted nature of autumn events^7^.

Understanding how autumn phenology respond to climate change are faced with two major challenges.

First, observational studies of autumn phenology have difficulties in defining events, treating autumn phenophases as multiple-day events, and standardizing methodologies^7^. The definition of autumn phenophases are based on subjective observations or descriptions of leaf colors^8^. Also, it is inaccurate to assign single dates to autumn phenophases since autumn leaf senescence, ranging from the date of first leaf changing color to the date of 100% abscission, are protracted and asynchronous. In addition, most papers do not describe the methods adequately, making it difficult to compare records across studies^9^. It is greatly desired to develop new strategy for capturing autumn senescence of plant appropriately.

Second, current understanding of the environmental control of autumn senescence mainly comes from endogenous hormones, which play important roles in regulating developmental processes involved in plant responses to changes in climate^10^. Change of the climate factors affect the synthesis and/or signaling pathways of hormones, thus further regulate the expression of senescence-related genes, which in turn appears to affect the timing of leaf senescence. Each kind of hormone could respond to various environmental events and participate in multiple regulatory pathways in a complex manner, but most of which are not understood^11^. Evidence suggests cytokinins (CKs), abscisic acid (ABA), and jasmonates (JAs) participate in mediating leaf senescence and plant responses to some weather stresses. CKs regulate cell proliferation and participate in plant responses to drought, and have been known as senescence-delaying hormones^12^. ABA is another key plant hormone mediating plant response to climate factors^13^. Exogenous application of ABA promotes leaf abscission and senescence, but the role of endogenous ABA in senescence has not been clearly defined^14^. JAs participate in plant response to cold temperature and influence many developmental processes including leaf senescence^15^. Methyl jasmonate (MeJA) and its precursor jasmonate (JA) have been known to promote senescence in *Avena sativa* leaves^16^. Auxins are involved in the senescence process, but the role of auxins in leaf senescence remains elusive, especially due to its involvement in various aspects of plant development including cell proliferation and elongation^17^. The role of gibberellins (GAs) and brassinosteroids (BRs) in senescence regulation and environmental responses are not fully understood^18^. In addition, the understanding of hormones in controlling senescence and responses to climate change has mostly based on behavior of *Arabidopsis*, a favorite model plant for biological study. It should be noted that *Arabidopsis*, as a monocarpic plant, has a different senescence character from that of perennial plants like deciduous trees^19^. Thus, the findings in *Arabidopsis* might not reveal some of the mechanisms involved in senescence and plant responses to climate factors of deciduous trees.

Therefore, the objective of this study is to provide a method that define autumn senescence appropriately as well as investigate the mechanism behind autumn phenological responses to climate change through hormone regulation for deciduous tree species. In this study, behaviors of four types of deciduous tree species (*Salix babylonica*, *Ginkgo biloba*, *Acer mono*, *Cotinus coggygria*) were selected as the subject of study, since they are the dominant species in China with special manifestation of coloring. Chlorophyll, as a well-established senescence marker, was used to quantitatively measure the state of autumn senescence^19^. The content of chlorophyll in in leaf tissues during the entire autumn senescence processes were monitored to provide a standardized and comparable method on defining autumn phenological events. The profile of nine major endogenous hormones, including indole-3-acetic acid (IAA), indole-3-propionic acid (IPA), zeatin riboside (ZR), dihydrozeatin riboside (DHZR), gibberellins (GA_3_, GA_4_), abscisic acid (ABA), MeJA, and brassinosteroid (BR) during the entire autumn senescence processes were established. Important climate factors (temperature, photoperiod, precipitation, humidity) affecting the timing of autumn senescence were firstly determined. Major plant hormones sensitive to climate factors were also identified. Then, hormonal regulation networks towards autumn senescence were investigated by system analysis. Finally, the mechanism of climate factors affecting the timing of autumn senescence through endogenous hormone was elucidated.

## Result and Discussion

### Interannual climate variation

**Figure 1:**
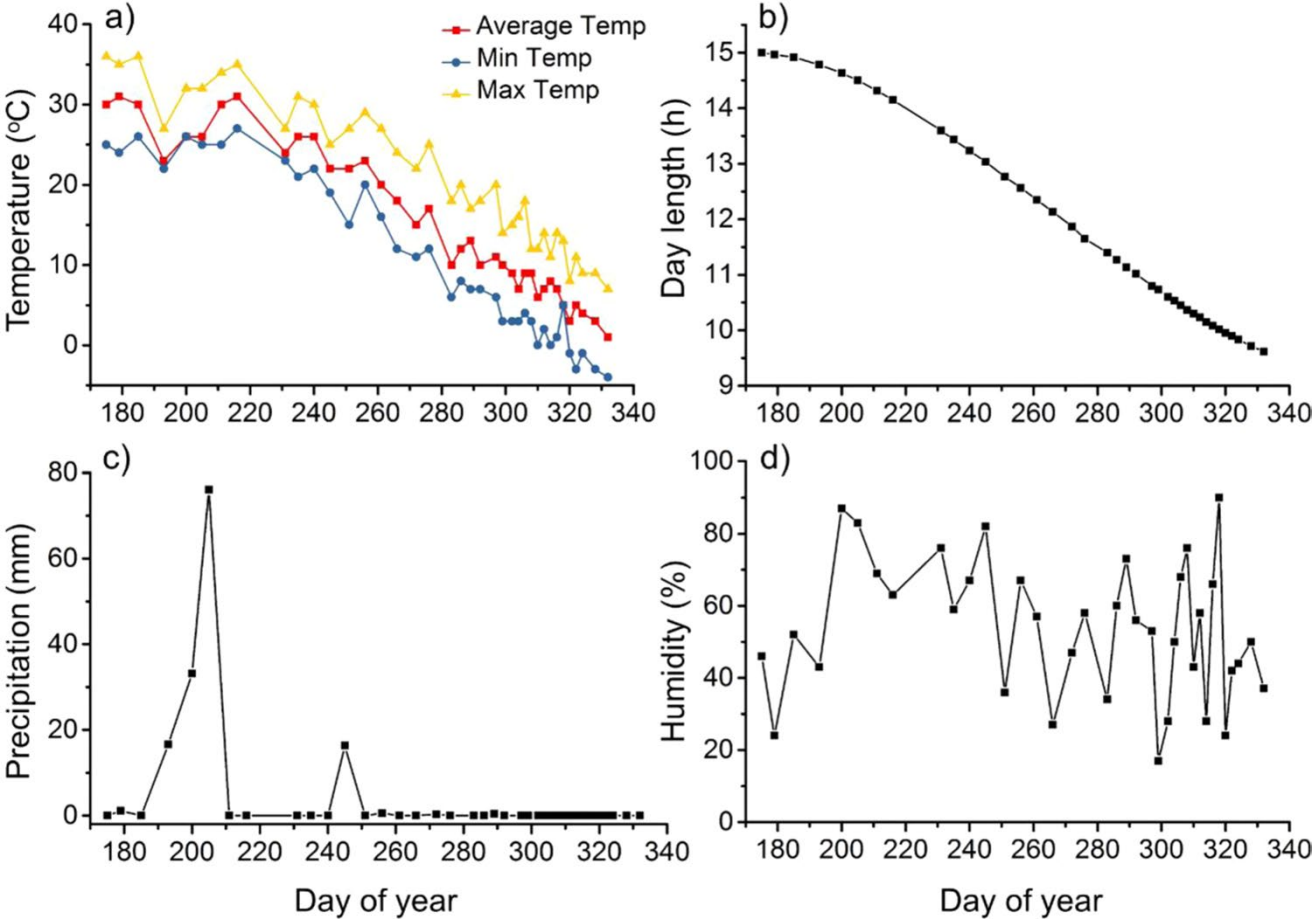
Change of temperature (**a**), day length (**b**), precipitation (**c**), and humidity (**d**) during autumn senescence.

During the study period, daily weather data including average temperature (Fig. 1a), day length (Fig. 1b), precipitation (Fig. 1c), and humidity (Fig. 1d) were collected on China Meteorological Administration (www://cdc.cma.gov.cn/). According to literature, cooling temperature and shortened day length are climate factors which trigger senescence^18^. Thus, samples were collected from the day with almost the highest temperature and longest day length of the year, 24^th^ Jun. 2018, to 28^th^ Sep. 2018, last leaf drop of *Salix babylonica*.

### Definition of autumn events

**Figure 2:**
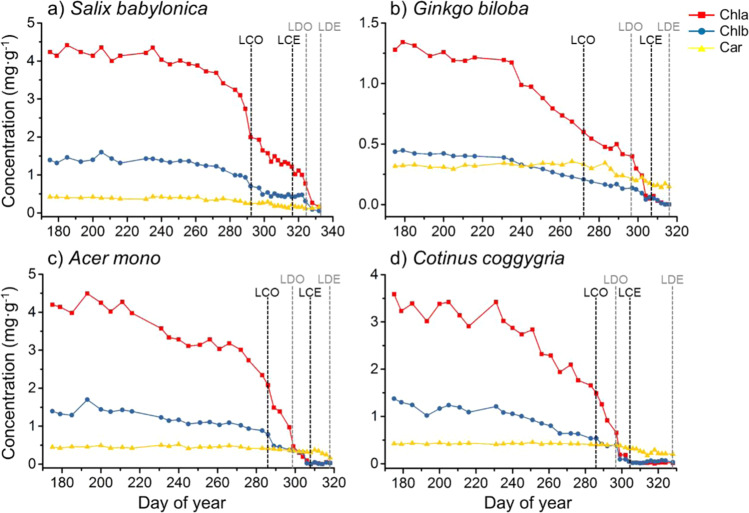
Phytochrome content in leaf tissue against different autumn phenological stages. (**a**) *Salix babylonica* (**b**) *Ginkgo biloba* (**c**) *Acer mono* (**d**) *Cotinus coggygria*. * Abbreviations: LCO, leaf coloration onset; LCE, leaf coloration end; LDO, leaf drop onset; LDE, leaf drop end; Chla, chlorophyll A; Chlb, chlorophyll B; Car, carotenoids.

Chlorophyll, as a well-established senescence marker, was used to properly quantify the state of autumn senescence. The concentration fluctuations of three major phytochrome (Chla, Chlb, and Car) in leaf tissue of *Salix babylonica* (Fig. 2a)*, Ginkgo biloba* (Fig. 2b)*, Acer mono* (Fig. 2c), and *Cotinus coggygria* (Fig. 2d) during the entire autumn phenological processes were studied. Similar trend was found among the four species, the level of Chl began to drop once senescence started while the level of Car remains stable until the end of leaf coloration. Chl degrades during senescence, while Car are relatively stable, which results in the change of leaf color.

Chla have higher concentration than Chlb in leaf tissue, so it was chosen as the indicator of autumn senescence. Beginning of leaf coloring could be observed when about 50% of the Chl was lost. Except for *Salix babylonica* (leaf color mainly stays green), end of leaf coloring could be observed when Chl content was close to 0. To eliminate uncertainty from autumn event determined by observation and to provide a unified method, beginning of leaf coloration could be re-defined as when about 50% of the Chl was lost. End of leaf coloration could be re-defined as when about 95% of the Chl was lost.

### Profile of endogenous hormone

**Figure 3:**
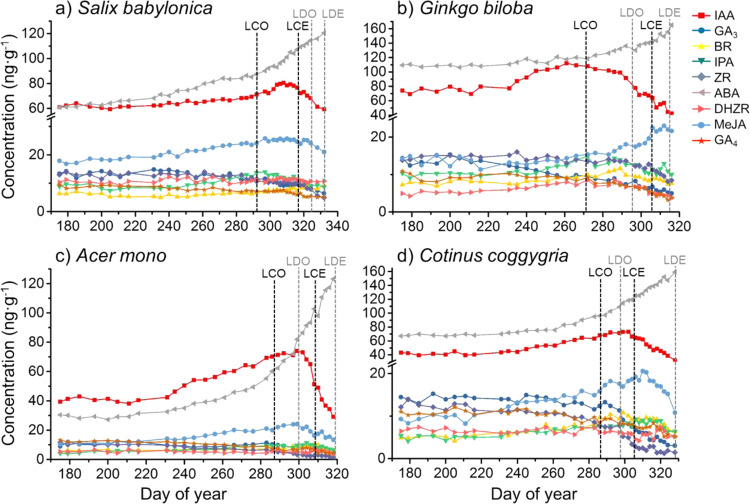
Hormone content in leaf tissue against different autumn phenological stages. (**a**) *Salix babylonica* (**b**) *Ginkgo biloba* (**c**) *Acer mono* (**d**) *Cotinus coggygria*. * Abbreviations: LCO, leaf coloration onset; LCE, leaf coloration end; LDO, leaf drop onset; LDE, leaf drop end; IAA, indole-3-acetic acid; IPA, indole-3-propionic acid; ZR, zeatin riboside; DHZR, dihydrozeatin riboside; GA_3_, gibberellin 3; GA_4_, gibberellin 4;ABA, abscisic acid; MeJA, methyl jasmonate; BR, brassinosteroid.

Endogenous hormones are involved in autumn phenological processes and their concentrations are dynamic. The concentrations and the fluctuation of IAA, IPA, ZR, DHZR, GA_3_, GA_4_, ABA, MeJA, and BR in leaf tissue of *Salix babylonica* (Fig. 3a)*, Ginkgo biloba* (Fig. 3b)*, Acer mono* (Fig. 3c), and *Cotinus coggygria* (Fig. 3d) were studied to better understand the variation pattern of endogenous hormones in leaf tissues at different senescence stages. Throughout the entire autumn phenological processes, the concentration of GA_3_, GA_4_, and ZR decreased, while ABA concentration increased. IAA accumulated in the early stage of senescence, but started to decrease in the middle of senescence. The content of MeJA mainly increased, except for the end of leaf fall. Although the level of DHZR, IPA, and BR remained relatively stable during autumn senescence, the increasing of BR content in three species (except for *Acer mono*) could be observed.

Molecular biology studies reported similar result that ABA initiates and promotes senescence^20^. Also, the decrease of ZR was the first sign of senescence^17^. Studies also pointed out the accumulation of IAA at the early senescence stage is to prevent leaf fall, while the decrease in IAA content at final senescence stage is to trigger leaf fall^21,22^. Similar result was found that JAs mainly promotes senescence in the beginning of senescence^23^. BRs also had positive effect on senescence^18^.

### Relationship between autumn senescence and climate factors

Phenological studies based on observation have low time resolution, which makes the proper quantification of autumn phenophases difficult. To monitor autumn phenophases accurately, we proposed an experimental method based on Chla detection instead of observation.

**Figure 4:**
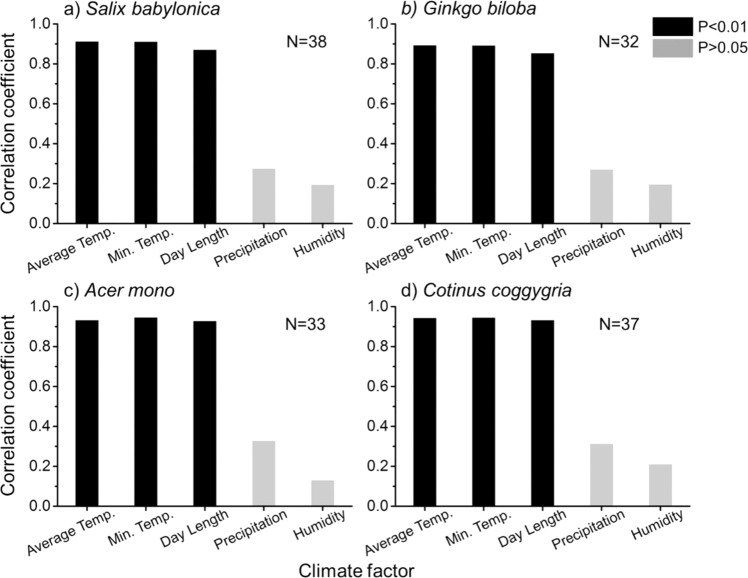
Correlation coefficient between climate factors (average daily temperature, minimum daily temperature, day length, precipitation, humidity) and Chla. (**a**) *Salix babylonica* (**b**) *Ginkgo biloba* (**c**) *Acer mono* (**d**) *Cotinus coggygria*.

First, correlation analyses between senescence (Chla content) and climate factors (temperature, day length, precipitation, humidity) were performed (Fig. 4). Chla content in leaf tissue of the four species all exhibited strong positive correlation with temperature (average daily temperature, minimum daily temperature) and day length, indicating that lowering temperature and shortened day length would result in a decrease in Chla which promote autumn phenological processes. Temperature and day length are the major climate factors affecting autumn phenological processes (R > 0.75, P < 0.01). Among the four studied species, temperature impacts more on senescence than day length. For *Acer mono* and *Cotinus coggygria*, minimum daily temperature has greater effect on senescence than average daily temperature.

This result corresponds well with phenological studies of deciduous trees based on years of observations, that temperature is the most important factor impacting autumn phenology^24^.

**Figure 5:**
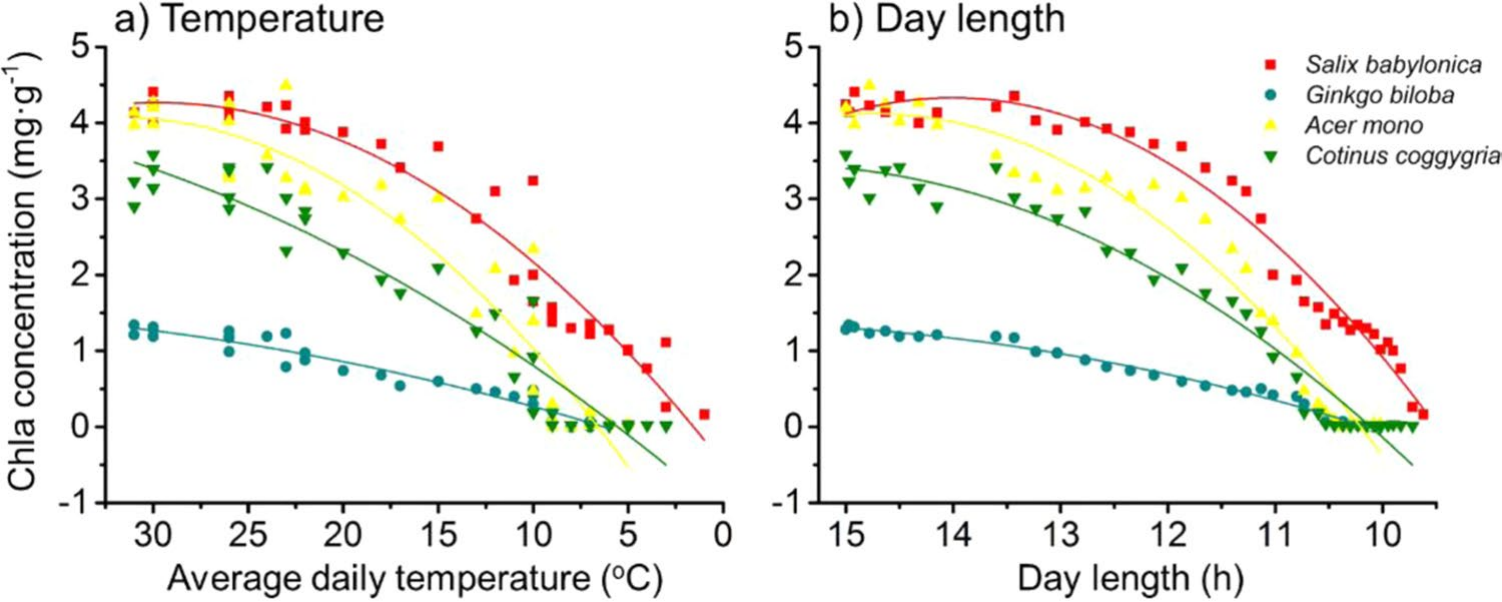
Relationship between major climate factors ((**a**) temperature (**b**) day length) and Chla.

Then, Chla content in leaf tissue were plotted along with average daily temperature (Fig. 5a) and day length (Fig. 5b). A nonlinear relationship between Chla content and average daily temperature/day length until the end of leaf drop (since sampling date) was manifested. Basically, low temperature and short day length tends to accelerate Chla lose which accelerate leaf senescence.

### Relationship between temperature/day length and endogenous hormone

**Figure 6:**
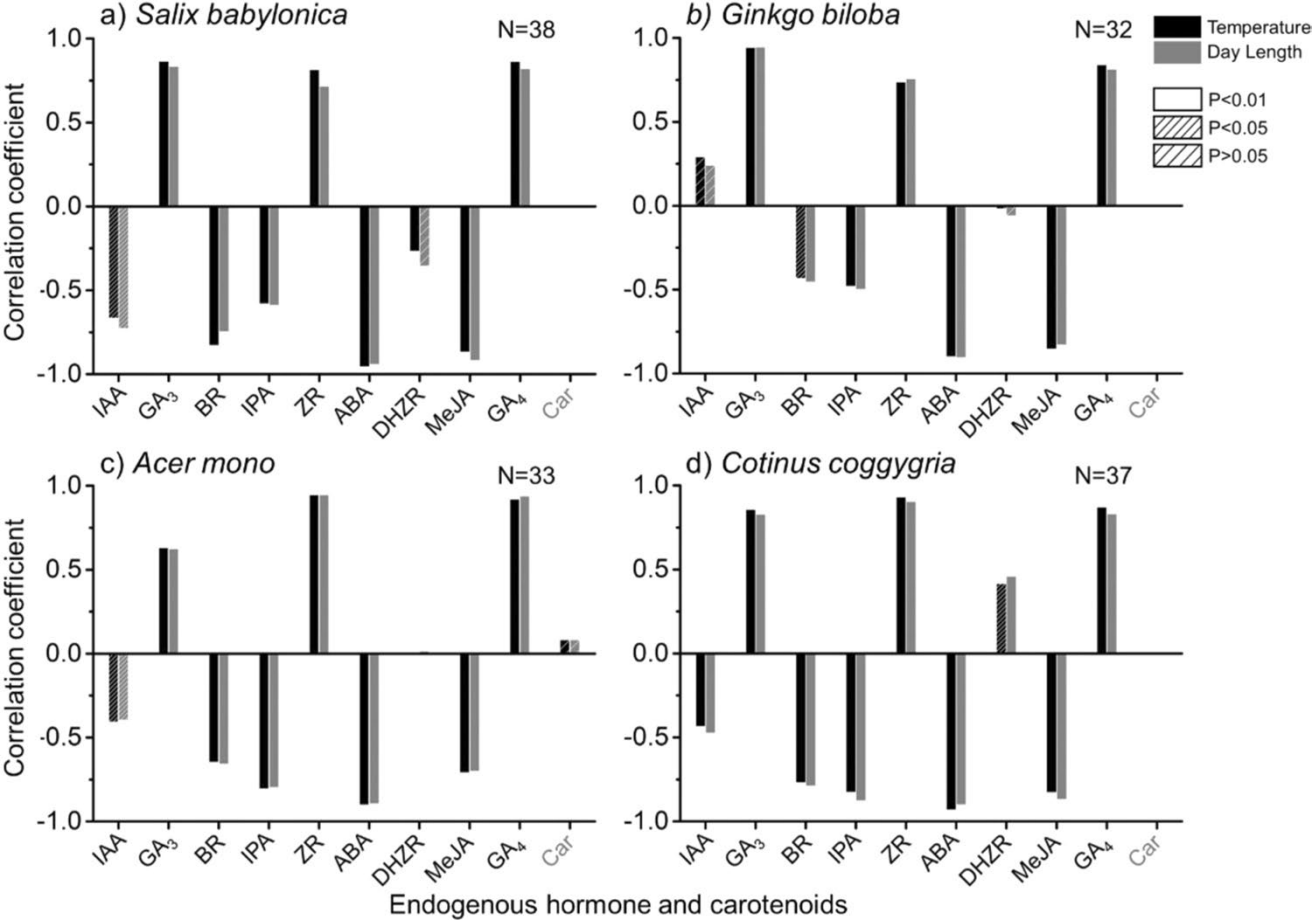
Correlation coefficient between major climate factors (black-temperature, grey-day length) and endogenous hormone. (**a**) *Salix babylonica* (**b**) *Ginkgo biloba* (**c**) *Acer mono* (**d**) *Cotinus coggygria*.

Changes in climate results in changes in different endogenous hormone levels. Alterations in plant hormone levels results in the changes in the expression of related genes and activation of plant responses, which trigger/regulate plant senescence. Correlation analyses were performed between climate factors (temperature, day length) and endogenous hormone (Fig. 6). ZR, ABA, MeJA, GA_3_, and GA_4_ exhibited strong correlation with temperature and day length (|R| > 0.75, P < 0.01), indicating that temperature and day length affect the timing of leaf senescence mainly through ZR, ABA, MeJA, GA_3_, and GA_4_ regulation. ZR, GA_3_, and GA_4_ exhibited positive correlation with temperature and day length, while ABA and MeJA exhibited negative correlation. This result suggests decreasing temperature and shortening day length would cause a reduction in ZR, GA_3_, and GA_4_ level (Fig. S1), as well as an increase in ABA and MeJA level (Fig. S2) to trigger/regulate senescence. IAA has different responses towards changes in temperature and day length among different species.

### Hormonal regulation of autumn senescence

First, correlation analysis was carried out on Chla and hormones to investigate the relationships in between. Correlation coefficients between endogenous hormones and Chla of *Salix babylonica* (Table S1), *Ginkgo biloba* (Table S2), *Acer mono* (Table S3), and *Cotinus coggygria* (Table S4) show the connection between different hormones. This result suggests hormones regulate the timing of autumn senescence systematically. The four studied species show similar results: ZR, GA_3_, and GA_4_ have positive correlation coefficients with Chla, indicating ZR, GA_3_, and GA_4_ exhibit inhibitory effect on senescence; ABA, BR, IPA and MeJA have negative correlation coefficients with Chla, indicating ABA, BR, IPA and MeJA act as enhancers of senescence. IAA has positive correlation coefficients with Chla in *Ginco Biloba* and *Acer Mono*, while it has negative correlation coefficients with Chla in *Salix Babylonica* and *Cotinus coggygria*. DHZR has positive correlation coefficients with Chla in *Ginco Biloba* and *Cotinus coggygria*, while it has negative correlation coefficients with Chla in *Salix Babylonica* and *Acer Mono*. These results suggest IAA and DHZR might delay senescence in some species while promote in other.

Second, a stepwise regression analysis of hormone regulation on Chla was conducted. Multivariate correlation equation, correlation coefficient (R), determinant coefficient (R^2^), and residual (e) between Chla and endogenous hormones are listed in Table. S5. The multivariate correlation equation of *Acer Mono* is the best fit for the data (with smallest residue), and was chosen as the formula of endogenous hormones to Chla. [Chla] = 7.320 − 0.053[ABA] + 0.039[ZR] − 0.034[IAA]. In that formula, Chla is significantly correlated with ABA, ZR, and IAA. Partial correlation coefficients are −1.001**, 0.281**, and 0.075* respectively(*P < 0.05, **P < 0.01). ABA, ZR and IAA might have a direct effect on autumn phenophases. Although other hormones have significant correlation with Chla, partial regression coefficient is not significant, which means these hormones might affect autumn phenophases indirectly. So we conduct a stepwise regression analysis of ABA, ZR and IAA with BR, IPA, DHZR, MeJA, GA_3_, and GA_4_, respectively. The regression equations are as follows:

$$[{\rm{ABA}}]=172.25-3.193[{{\rm{GA}}}_{3}]-8.522[{{\rm{GA}}}_{4}],{\rm{R}}=0.932$$

$$[{\rm{ZR}}]=1.082-0.542[{\rm{IPA}}]+3.193[{{\rm{GA}}}_{3}]+0.637[{{\rm{GA}}}_{4}],{\rm{R}}=0.966$$

$$[{\rm{IAA}}]=-25.99+3.654[{\rm{MeJA}}]+2.041[{{\rm{GA}}}_{4}],{\rm{R}}=0.942$$

Based on that, endogenous hormones are classified into three groups: (1) ABA, ZR, and IAA (affect senescence directly); (2) GA_3_, GA_4_, MeJA, and IPA (affect senescence indirectly through ABA, IAA, and ZR); (3) DHZR and BR (no direct effect on ABA, ZR, and IAA).

**Figure 7:**
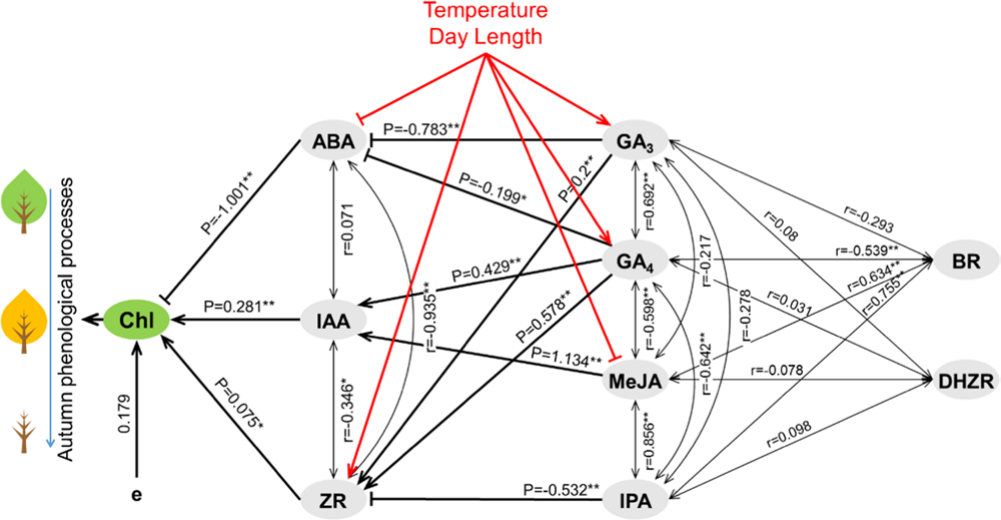
Hormone regulation network of autumn senescence towards climate factors in deciduous trees. P-path coefficient, R-relative coefficient, e-other path. Low temperature and short day length could result in the decrease of ZR level and the increase of ABA level, which directly trigger/promote senescence. Meanwile, low temperature and short day length could cause the decrease of MeJA level and the increase of GA_3_ and GA_4_ level, which regulate the timing of autumn senescence indirectly through ZR, ABA, and IAA. **- remarkable at 0.01 level, *-remarkable at 0.05 level.

Third, possible pathways and relative importance of ABA, ZR and IAA on autumn senescence regulation were analyzed. Also, the path analysis of hormones on ABA, ZR and IAA was carried out by SPSS 22.0. The results were summarized as a pathway network of hormonal systems regulating the timing of autumn senescence (Fig. 7). Path coefficient (P) between Chla and hormone or between two hormones is partial correlation coefficient from stepwise regression analysis. The path of hormone regulating autumn senescence were show in Table. S6–S12. Basically, ABA could promote autumn senescence both directly, and indirectly by antagonizing the delayed action of ZR. ZR could delay autumn senescence both directly, and indirectly by increasing IAA and decreasing ABA level. IAA could delay autumn senescence directly, meanwhile promote senescence through ABA and ZR indirectly.

This result also corresponds well with plant physiology studies that exogenous addition of ABA could reduce the level of CKs while accelerate leaf senescence^25^. Also, GAs and CKs have positive synergistic effects on leaf senescence^26^. Molecular biology study pointed out similar result that GAs could not regulate senescence directly, instead indirectly by antagonizing the inhibitory effect of ABA. The role of auxins in senescence regulation is still controversial^27^. Our result shows the possibility that IAA could both promote and inhibit senescence through different pathways. Although JAs were found to have positive effect on senescence, the mechanism of JAs regulating senescence is complicated and still unclear. Our result shows the possibility that MeJA could regulate senescence through IAA^28^.

### Regulation of senescence-timing by climate factors of deciduous trees

Figure 7 shows the overview of regulation of autumn senescence timing by temperature and day length in deciduous trees. Temperature and day length affect the timing of leaf senescence mainly through ZR, ABA, MeJA, GA_3_, and GA_4_ regulation (|R | > 0.75, P < 0.01). Decreasing temperature and shortening day length would cause a reduction of ZR, GA_3_, and GA_4_ level (R > 0), as well as an increase of ABA and MeJA level (R < 0) in leaf tissue. The decrease of ZR level and the increase of ABA level would directly trigger/promote senescence. Meanwhile, the decrease pf MeJA level and the increase of GA_3_ and GA_4_ level could affect the timing of autumn senescence indirectly through ZR, ABA, and IAA.

## Conclusion

A method to monitor autumn events based on measuring Chl content in leaf tissue was described in this study. An accurate and standardized definition of autumn phenophases (beginning of leaf coloring, end of leaf coloring) could be given according to Chl content in leaf tissue. Beginning of leaf coloring could be observed when about 50% of the Chl was lost. End of leaf coloring could be observed when about 95% of the Chl was lost. This method could achieve better accuracy than observation, and has the potential to be used as a standard method for autumn phenology study.

Based on profiling nine major plant hormone in leaf tissue during the entire autumn phenological processes, possible regulatory pathways between climate factors and plant senescence was established. Low temperature and short day length could result in the decrease of ZR level and the increase of ABA level in leaf tissue, which directly trigger/promote senescence. Meanwhile, low temperature and short day length could cause the decrease of MeJA level and the increase of GA_3_ and GA_4_ level in leaf tissue, which affect the timing of autumn senescence indirectly through ZR, ABA, and IAA regulation. This study improves the understanding of how plant responses to climate change. However, for the further understanding of regulation on autumn phenology, some important clues obtained by transcriptome and other omics are needed.

## Methods

### Time and area of study

All data were collected from 24^th^ Jun. 2018 (leaf senescence onset), to 28^th^ Nov 2018 (leaf drop end). Plant samples were collected in Wanliu, Beijing, China, which extends across 39.98°–39.99°N, 116.29°–116.31°E. This area lies on typical monsoon climate with a mean annual precipitation between 500 and 600 mm (1981–2010) and a mean annual temperature of 12.9 °C. The coldest month was January, while the warmest month was July.

### Sample collection and pheonology observation

Four dominate deciduous tree species (Table 1), including *Salix babylonica, Ginkgo biloba, Acer mono, Cotinus coggygria*, were selected for this study. They are the dominant species in China which can reflect autumn phenological changes over large regions and have special manifestation of coloring: (1) *Salix babylonica* is one of the last leaf-coloring species in China am the leaf color mainly remains green during senescence; (2) leaf color of *Ginkgo biloba* changes from green to yellow; (3) leaf color of *Acer mono* changes from green to red; (4) leaf color of *Cotinus coggygria* changes from green to yellow to red. We observed leaf phenology for each individual, including LCO (leaf coloration onset), LCE (leaf coloration end), LDO (leaf drop onset), LDE (leaf drop end) during sample collection. Observation and sample collection were conducted three times a week from 24^th^ Jun. 2018, to 28^th^ Sep. 2018, last leaf drop of *Salix babylonica*. A total number of 280 fresh leaf samples (76 for *Salix babylonica*, 64 for *Ginkgo biloba*, 66 for *Acer mono*, 74 for *Cotinus coggygria*) were collected for phytochrome analysis. A total number of 280 frozen leaf samples (76 for *Salix babylonica*, 64 for *Ginkgo biloba*, 66 for *Acer mono*, 74 for *Cotinus coggygria*) were collected for hormone analysis. Two parallel samples for each species were collected each time. For phytochrome analysis, at least three fresh leaves were collected in 4 °C ice box for each sample. For hormone analysis, three fresh leaves were collected for each sample and frozen in liquid nitrogen immediately, then stored at −80 °C for later analysis. Leaf samples were harvested in the afternoon.

**Table 1:** Summary of species and autumn phenological phases in 2018.

No.	Species	Family	Life Form	Color During Autumn Phenological Phases	Phases (Month/date)			
					LCO	LCE	LDO	LDE
1	*Salix babylonica*	Salicaceae	Tree	Green	10/19	11/12	11/20	11/28
2	*Ginkgo biloba*	Ginkgoaceae	Tree	Green-Yellow	10/4	11/4	10/24	11/12
3	*Acer mono*	Aceraceae	Tree	Green-Red	10/13	11/4	10/26	11/14
4	*Cotinus coggygria*	Anacardiaceae	Shrub/Small tree	Green-Yellow-Red	10/13	11/2	10/24	11/24

*LCO (leaf coloration onset), LCE (leaf coloration end), LDO (leaf drop onset), LDE (leaf drop end).

### Endogenous hormone analysis

#### Sample pre-treatment

The overall procedure was similar to the kit protocol^29,30^. Briefly, frozen leaf sample (0.2 g) was added into 4 mL of extraction buffer and ground into homogenate, then incubate at 4 °C for 6 h. The whole suspension was then transferred to a centrifuge tube and centrifuged at 3500 r·min^−1^ for 10 min. The supernatant was collected and mixed with 1 mL extraction buffer, and incubate at 4 °C for 1 h. Then the mixture was centrifuged at 3500 r·min^−1^ for 10 min. The supernatant was collected and purified with a C-18 column. Then the supernatant was dried using a nitrogen evaporator and re-dissolved in 200 μL of dilution buffer.

#### Hormone detection

Hormone working solutions (0–50 ng·mL^−1^), standards, and samples of 50 μL were prepared and transferred into 96-well plate. Standards were analyzed in four replicates. Two repetitions were performed. Add 50 μL of antibody into each well and incubate at 37 °C for 30 min. After washing the 96-well plate three times with 250 μL PBST buffer, add 100 μL of secondary antibody into each well and incubate at 37 °C for 30 min. Plates were then washed with PBST, which was followed by the addition of 200 μL substrate buffer. The reaction was stopped by adding 100 μL of stop buffer. Absorbance was read at 490 nm in the microplate reader.

The concentration (w/w) of hormone (IAA, IPA, ZR, DHZR, GA_3_, GA_4_, ABA, MeJA, BR) in leaf tissue (ng·g^−1^) could be calculated as:

$${\rm{C}}({\rm{ng}}\cdot {{\rm{g}}}^{-1})=\frac{{\rm{C}}({\rm{ng}}\cdot {{\rm{mL}}}^{-1})\times 0.2\,{\rm{mL}}}{0.2\,{\rm{g}}}$$

### Phytochrome analysis

#### Sample pre-treatment

Midlobe vein was first removed from fresh leaf sample. Then leaf sample of 0.2 g was mixed with arenaceous quartz, calcium carbonate, and 3 mL of 80% acetone. The mixture was ground into homogenate. Add 10 mL of 80% acetone into the homogenate and incubate in the dark for 10 min at room temperature. After filtration, add 80% acetone into the solution to 50 mL.

#### Phyrochrome detection

Absorbance was read at 663, 646, and 470 nm in the ultraviolet-visible spectrometer, with 80% acetone as blank solution. The concentration of Chla, Chlb, and Car in the solution (mg·L^−1^) could be calculated as:

$${{\rm{C}}}_{{\rm{a}}}=12.21{{\rm{A}}}_{663}-2.81{{\rm{A}}}_{646}$$

$${{\rm{C}}}_{{\rm{b}}}=20.13{{\rm{A}}}_{646}-5.03{{\rm{A}}}_{663}$$

$${{\rm{C}}}_{{\rm{c}}}=(1000{{\rm{A}}}_{470}-3.27{{\rm{C}}}_{{\rm{a}}}-104{{\rm{C}}}_{{\rm{b}}})/229$$

C_a_ represents the concentration of Chla. C_a_ represents the concentration of Chlb. C_c_ represents the concentration of Car. A_663_ represents the absorbance at 663 nm. A_646_ represents the absorbance at 646 nm. A_470_ represents the absorbance at 470 nm.

The concentration (w/w) of phyrochrome (Chla, Chlb, Car) in leaf tissue (mg·g^−1^) could be calculated as:

$${\rm{C}}({\rm{mg}}\cdot {{\rm{g}}}^{-1})=\frac{{\rm{C}}({\rm{mg}}\cdot {{\rm{L}}}^{-1})\times 0.05\,{\rm{L}}}{0.2\,{\rm{g}}}$$

#### Data analysis

The concentration of Chla was chosen as the indicator of leaf senescence. The content of hormones and phytochromes were used to demonstrate the correlation between hormones and Chla. Stepwise aggression analysis and pathway analysis of hormones affecting autumn phenological processes were carried out. Correlation analysis, including path way analysis, was conducted by SPSS 22.0 (Fig. S3).

### Chemicals and reagents

Endogenous hormone standards: indole-3-acetic acid (IAA), indole-3-propionic acid (IPA), zeatin riboside (ZR), dihydrozeatin riboside (DHZR), gibberellins (GA_3_, GA_4_), abscisic acid (ABA), jasmonic acid methyl ester (MeJA), and brassinosteroid (BR) were purchased from Olchemim Ltd. (Olomouc, Czech Republic).

*o*-phenylenediamine (OPD) were purchased from Sigma (St Louis, MO, USA). Methanol, acetone, hydrogen peroxide, and other chemicals were purchased from Beijing Chemical Reagents Company (Beijing, China). Ultra-pure water used throughout the study was purified with Milli-Q system (Milford, MA, USA). All chemical used were of analytical grade.

The following buffers were used:

PBS buffer (0.1 M phosphate buffer containing 0.9% NaCl, pH 7.5)

PBST buffer (PBS buffer with 0.1% (v/v) Tween-20)

Extraction buffer (80% methanol, 1 mmol·L^−1^ BHT)

Dilution buffer (PBST buffer with 0.5% (w/v) gelatin)

Citrate-phosphate buffer (0.01 M citric acid monohydrate, 0.03 M Na_2_HPO_4_, pH 5.5)

Substrate buffer (addition of 4 μL 30% H_2_O_2_ to 10 mL citrate–phosphate buffer containing 2 mg·mL^−1^ OPD)

Stop buffer (2 M H_2_SO_4_).

### Apparatus

Plant hormone kits were purchased from Beijing Beinongtianyi Biotechnology Limited (China). A microplate reader (Multiskan MK3, Thermo, Vantaa, Finland) and an UV-Vis spectrometer (PerkinElmer, Santa Clara, CA) were used.

## Supplementary information

Supplementary information.
