# Supplementary material for: Responses of Autumn Phenology to Climate Change and the Correlations of Plant Hormone Regulation
Source: Sci Rep. 2020 Jun 3;10:9039. doi: 10.1038/s41598-020-65704-8 (PMC7270090; doi:10.1038/s41598-020-65704-8)

**Responses of Autumn Phenology to Climate Change and the Correlations of Plant Hormone Regulation**

Zhang Shixi, Dai Junhu, Ge Quansheng*

Key Laboratory of Land Surface Pattern and Simulation, Institute of Geographic Sciences and Natural Resources Research, CAS, Beijing 100101, China

(email: geqs@igsnrr.ac.cn)

**Relationship between temperature/day length and plant hormone.**

**
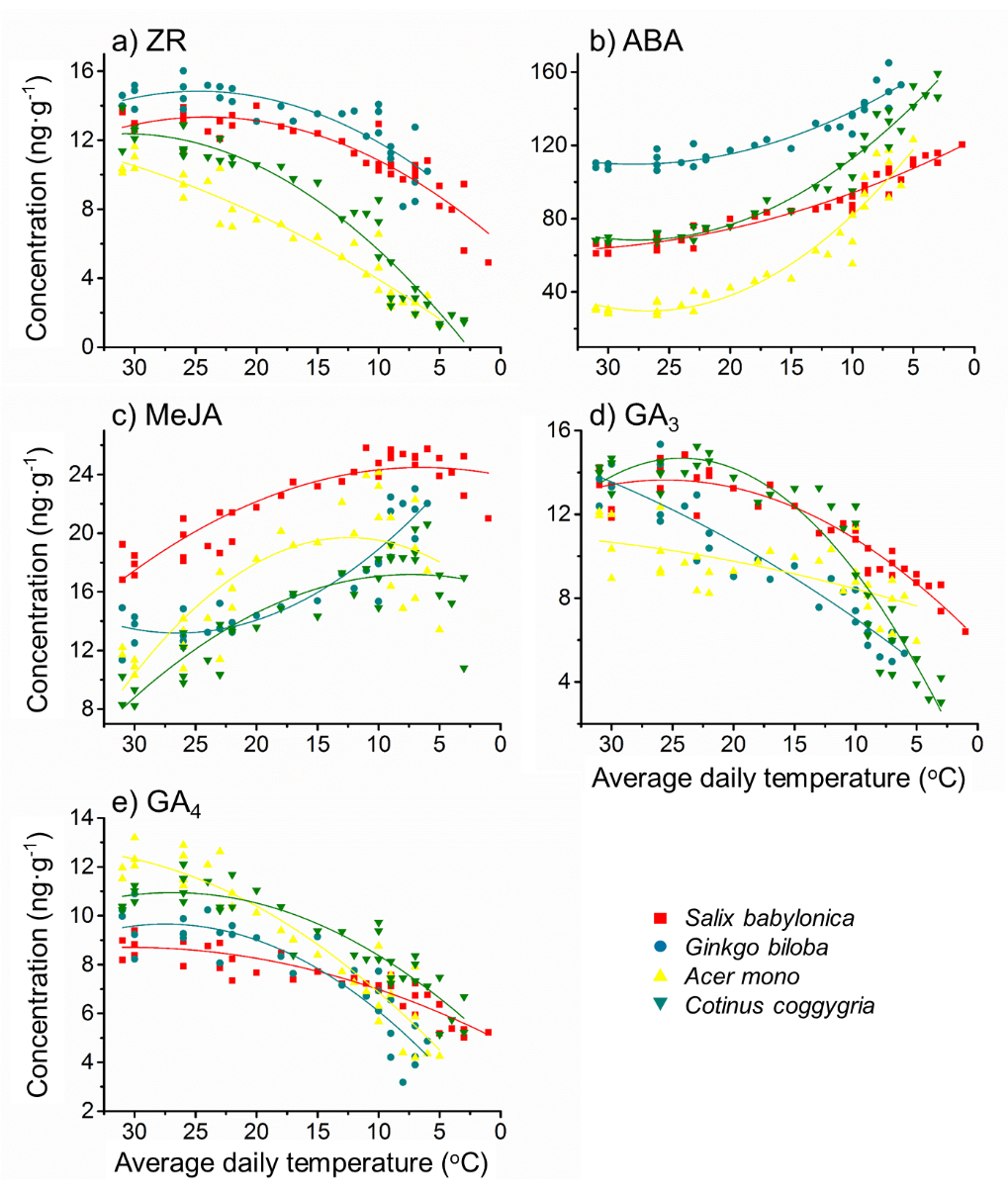
**

Figure S1. Relationship between temperature and endogenous hormone.


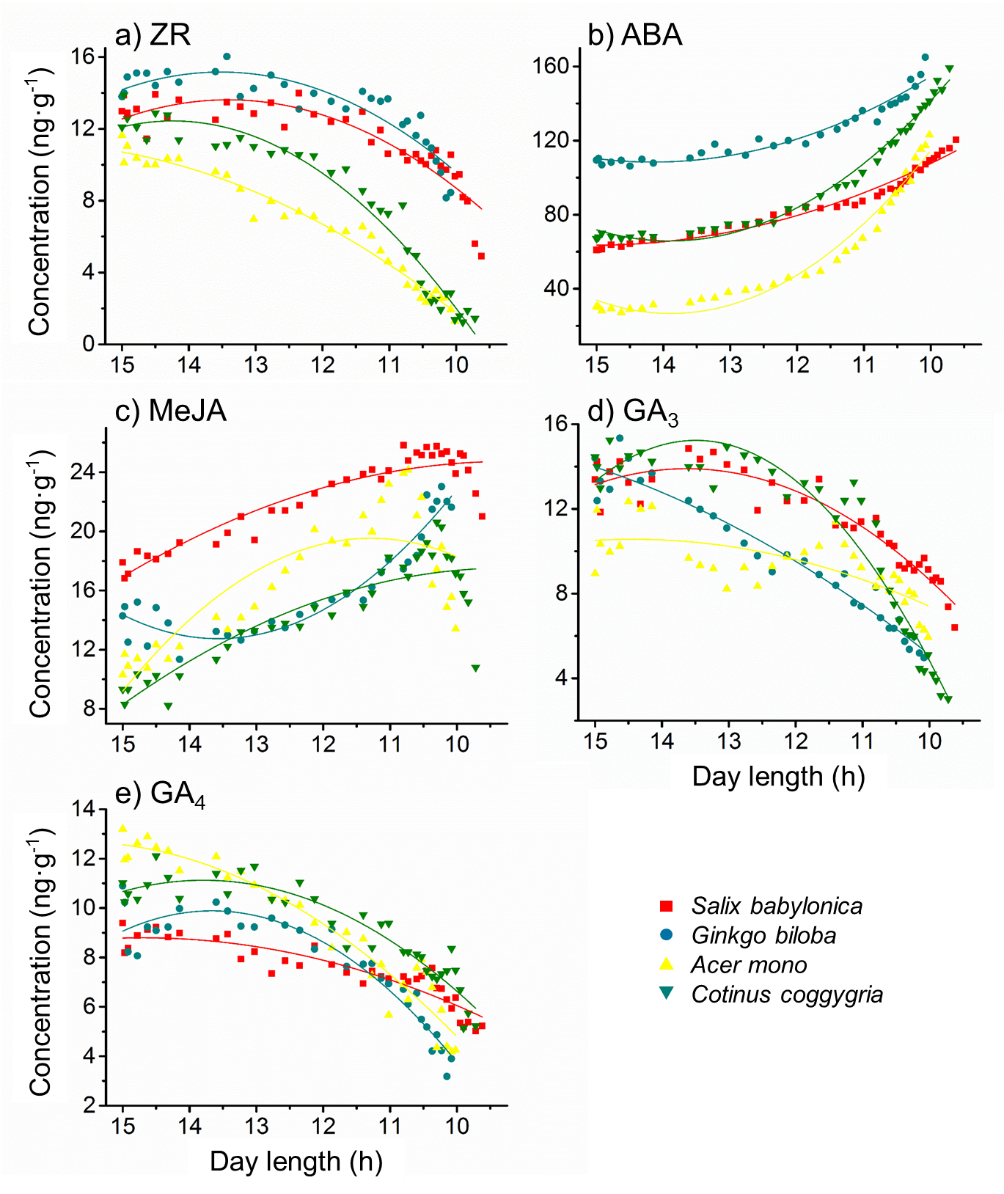


Figure S2. Relationship between day length and endogenous hormone.

**Hormonal regulation of autumn senescence.**

Correlation coefficients between endogenous hormones and Chla of *Salix babylonica* (Tab.S1), *Ginkgo biloba* (Tab. S2), *Acer mono* (Tab. S3), and *Cotinus coggygria* (Tab. S4).

**Table S1**

Correlation coefficients between endogenous hormones during autumn senescence processes of *Salix Babylonica*. n=38


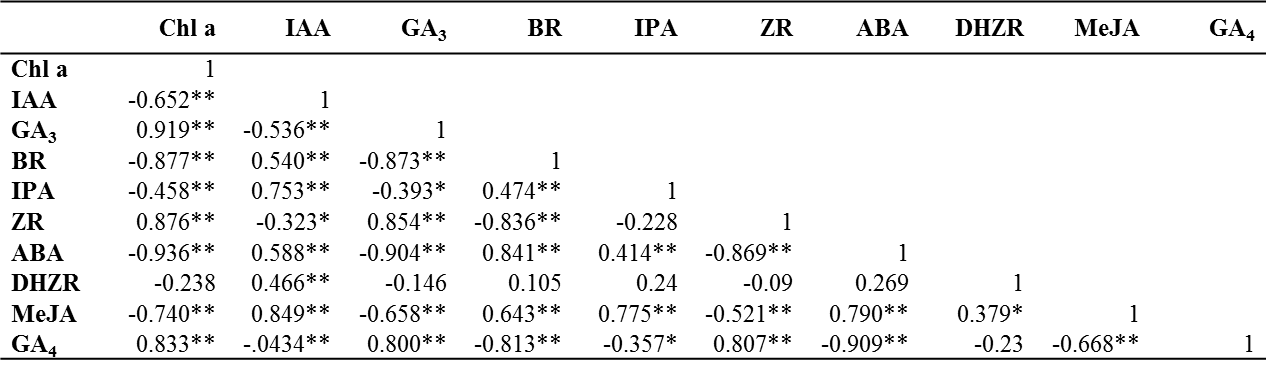


** stands for remarkable at 0.01 level, ** stands for remarkable at 0.05 level.

**Table S2**

Correlation coefficients between endogenous hormones during autumn senescence processes of *Ginco Biloba*. n=32


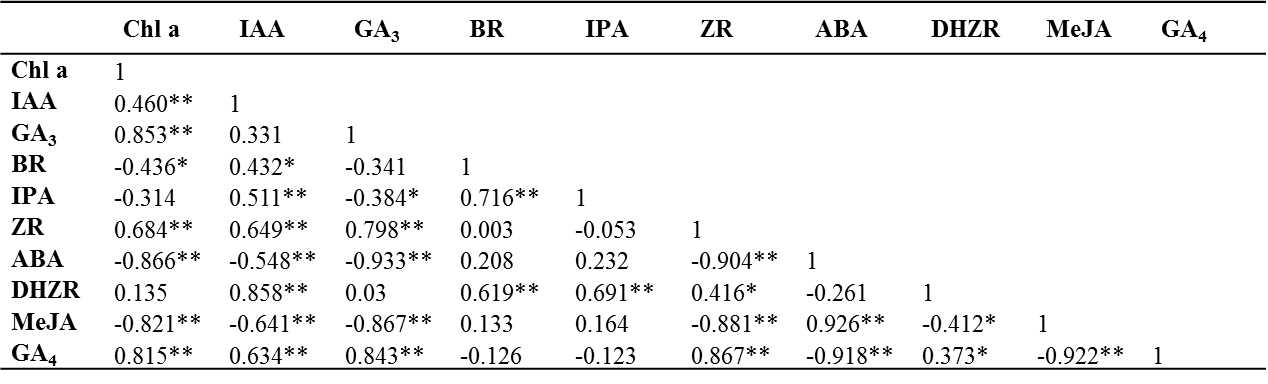


** stands for remarkable at 0.01 level, ** stands for remarkable at 0.05 level.

**Table S3**

Correlation coefficients between endogenous hormones during autumn senescence processes of *Acer Mono*. n=33


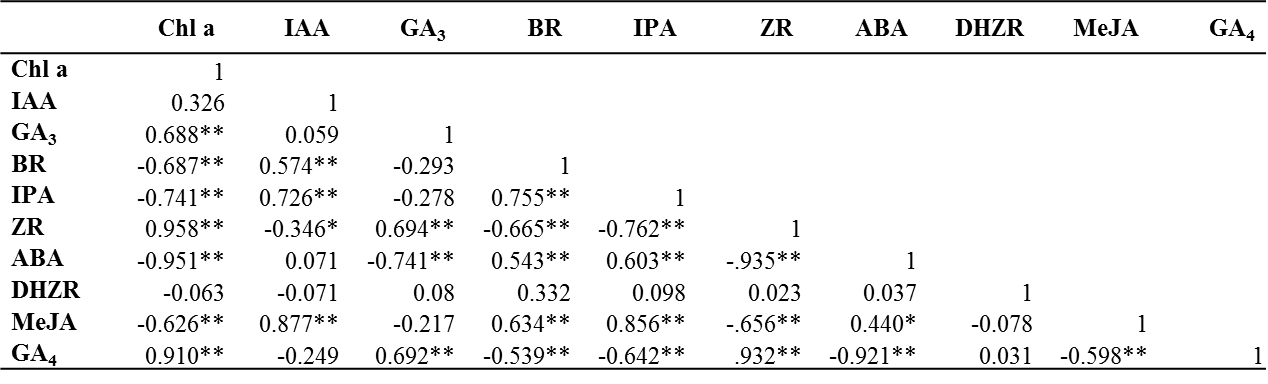


** stands for remarkable at 0.01 level, ** stands for remarkable at 0.05 level.

**Table S4**

Correlation coefficients between endogenous hormones during autumn senescence processes of *Cotinus coggygria*. n=37


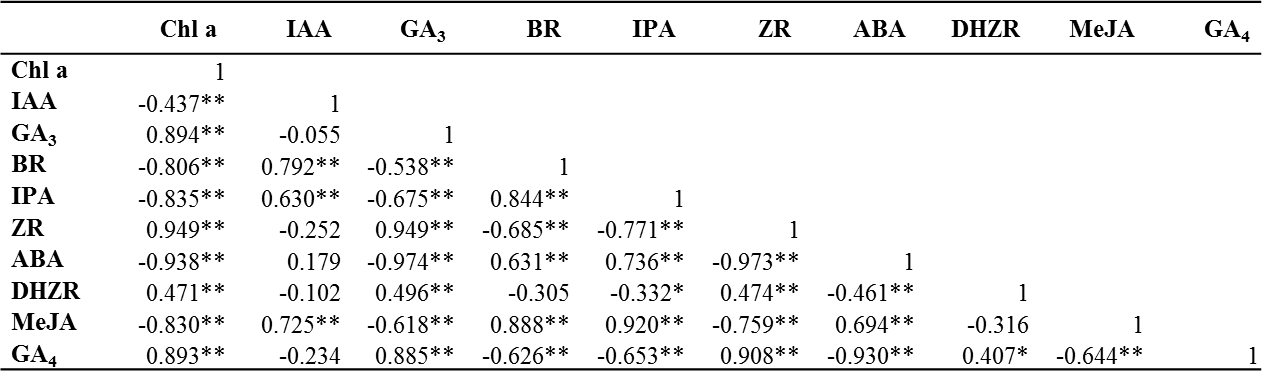


** stands for remarkable at 0.01 level, ** stands for remarkable at 0.05 level.

Stepwise regression analysis of hormone regulation on Chla was conducted. Multivariate correlation equation, correlation coefficient (R), determinant coefficient (R^2^), and residual (e) between Chla and endogenous hormones are listed in Tab.S5.

**Table S5**

Multivariate correlation equation between Chla and endogenous hormones


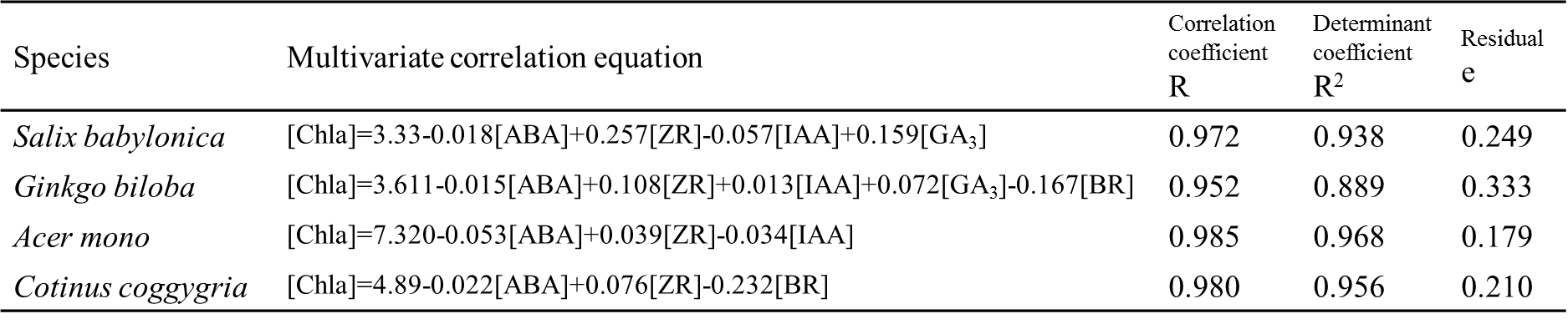


**Path analysis of endogenous hormone to Chla.**

ABA (Tab. S6), ZR (Tab. S7), and IAA (Tab. S8) have three paths that affect autumn senescence. GA_3_ (Tab. S9), GA_4_ (Tab. S10), MeJA (Tab. S11), and IPA (Tab. S12) have seven inter-connected paths that affect autumn senescence. Path coefficient could be calculated as:

Path coefficient from x_1_ to y (P_1y_)=partial correlation coefficient between x_1_ and y

Path coefficient from x_2_ to x_1_ (P_21_)=partial correlation coefficient between x_2_ and x_1_

Path coefficient from x_2_ to y (P_2y_)= P_21_×P_1y_

Path coefficient from x_3_ to y (P_2y_)= r_23_×P_21_×P_1y_

*y: Chla. x_1_: ABA, ZR, and IAA (affect senescence directly). x_2_: GA_3_, GA_4_, MeJA, and IPA (affect senescence indirectly through ABA, IAA, and ZR). x_3_: DHZR and BR (no direct effect on ABA, ZR, and IAA). r_23_: correlation coefficient between x_2_ and x_3_. Correlation coefficient and partial correlation coefficient was calculated by SPSS 22.0. (Fig. S3)


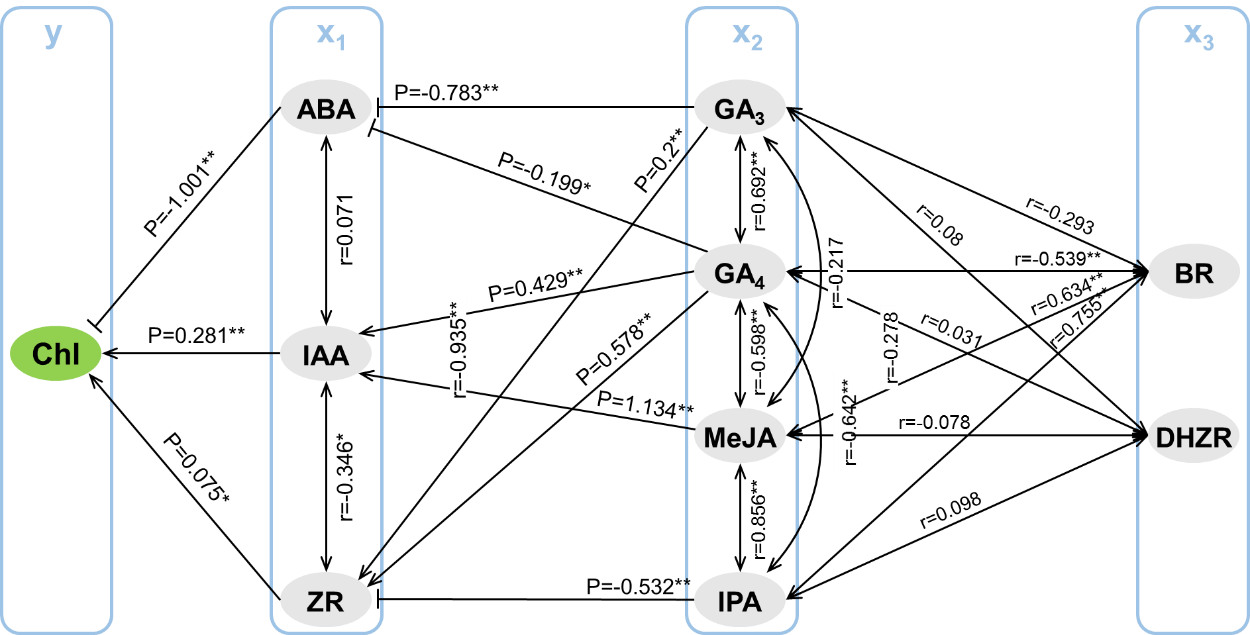


Fig. S3 Categorization of hormones

Path delaying autumn phenophases has negative path coefficient, whereas path promoting autumn phenophases has positive path coefficient.

**Table S6**

Path analysis of ABA to leaf senescence


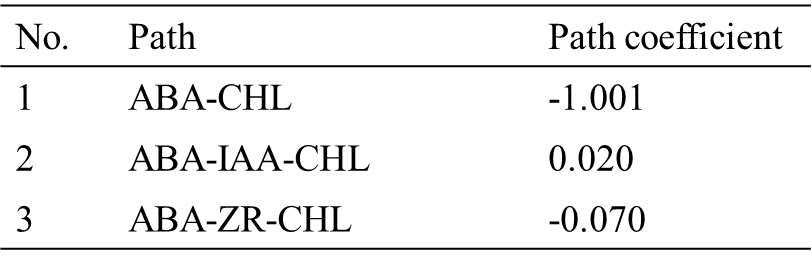


**Table S7**

Path analysis of ZR to leaf senescence


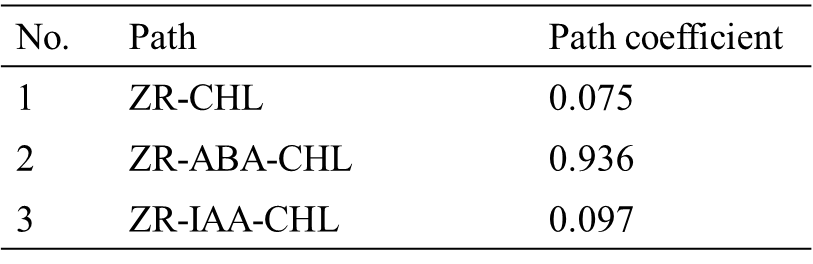


**Table S8**

Path analysis of IAA to leaf senescence


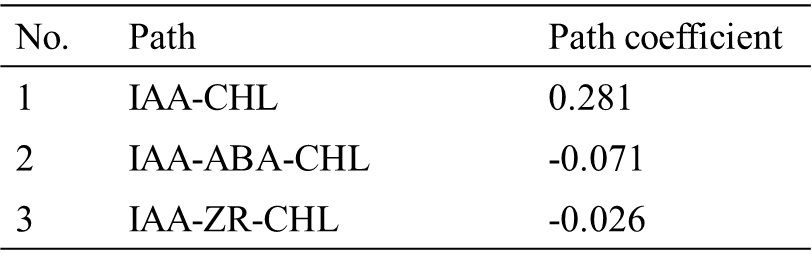


**Table S9**

Path analysis of GA_3_ to leaf senescence


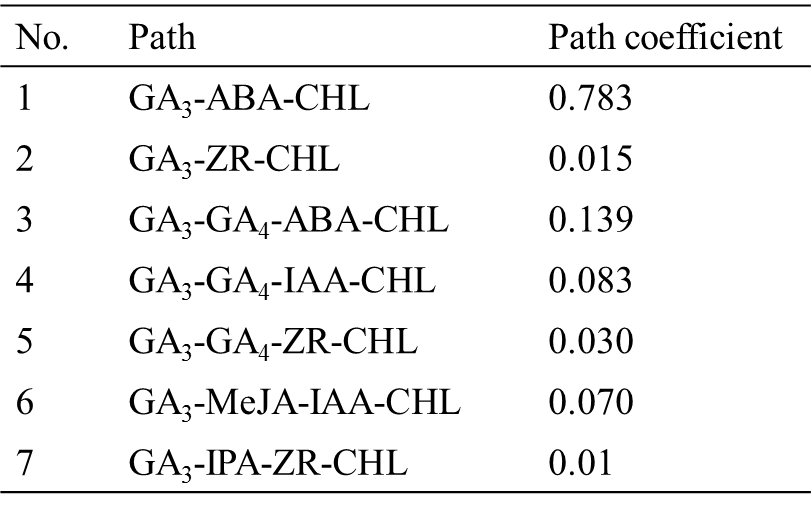


**Table S10**

Path analysis of GA_4_ to leaf senescence


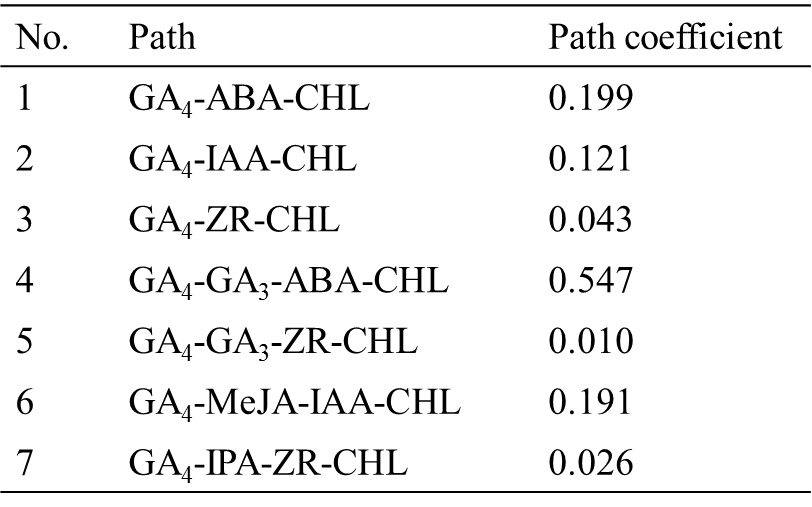


**Table S11**

Path analysis of MeJA to leaf senescence


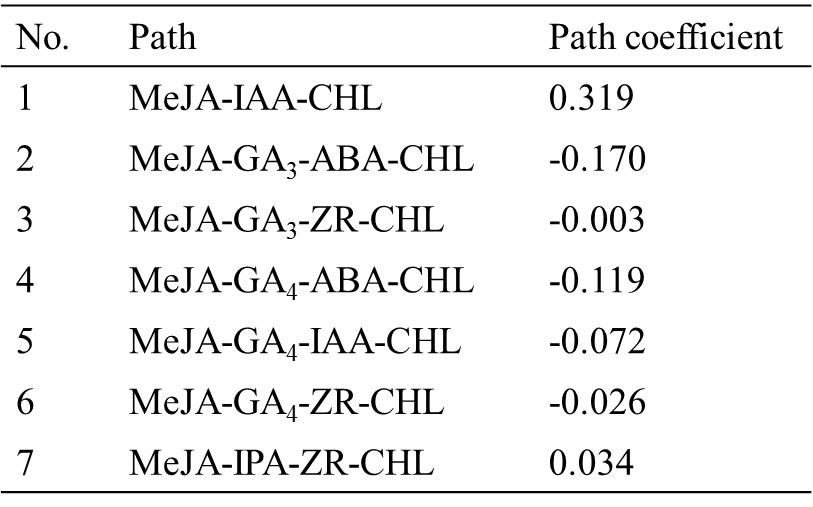


**Table S12**

Path analysis of IPA to leaf senescence


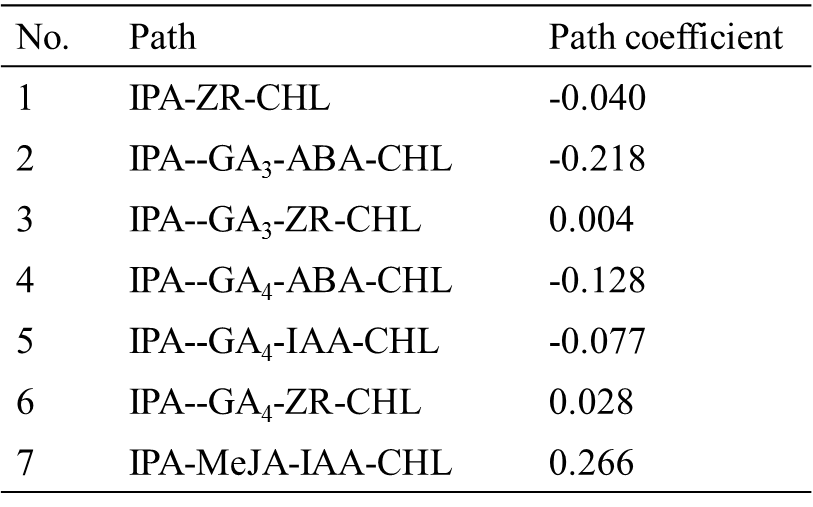

Supplement: Supplementary file 1 — Supplementary information. [file 41598_2020_65704_MOESM1_ESM.docx]
